# AI as an intervention: improving clinical outcomes relies on a causal approach to AI development and validation

**DOI:** 10.1093/jamia/ocae301

**Published:** 2025-01-07

**Authors:** Shalmali Joshi, Iñigo Urteaga, Wouter A C van Amsterdam, George Hripcsak, Pierre Elias, Benjamin Recht, Noémie Elhadad, James Fackler, Mark P Sendak, Jenna Wiens, Kaivalya Deshpande, Yoav Wald, Madalina Fiterau, Zachary Lipton, Daniel Malinsky, Madhur Nayan, Hongseok Namkoong, Soojin Park, Julia E Vogt, Rajesh Ranganath

**Affiliations:** Department of Biomedical Informatics, Columbia University, New York, NY 10032, United States; BCAM—Basque Center for Applied Mathematics, Bilbao 48009, Spain; IKERBASQUE—Basque Foundation for Science, Bilbao 48009, Spain; Julius Center for Health Sciences and Primary Care, University Medical Center Utrecht, Utrecht 3584 CX, The Netherlands; Department of Biomedical Informatics, Columbia University, New York, NY 10032, United States; Department of Biomedical Informatics, Columbia University, New York, NY 10032, United States; Division of Cardiology, Columbia University, New York, NY 10032, United States; Department of Electrical Engineering and Computer Sciences, University of California, Berkeley, Berkeley, CA 94720, United States; Department of Biomedical Informatics, Columbia University, New York, NY 10032, United States; Department of Anesthesiology and Critical Care Medicine, Johns Hopkins University School of Medicine, Baltimore, MD 21287, United States; Population Health and Data Science, Duke Institute of Health Innovation, Durham, NC 27701, United States; Department of Computer Science and Engineering, University of Michigan, Ann Arbor, Ann Arbor, MI 48109, United States; Department of Medicine, NYU Grossman School of Medicine, New York, NY 10016, United States; Center for Data Science, New York University, New York, NY 10011, United States; College of Information and Computer Sciences, University of Massachusetts, Amherst, Amherst, MA 01003, United States; Department of Machine Learning, Carnegie Mellon University, Pittsburgh, PA 15213, United States; Department of Biostatistics, Columbia University, New York, NY 10032, United States; Department of Population Health and Urology, NYU Grossman School of Medicine, New York, NY 10016, United States; Division of Decisions, Risk, and Operations, Columbia Business School, New York, NY 10027, United States; Department of Biomedical Informatics, Columbia University, New York, NY 10032, United States; Department of Computer Science, ETH Zurich, Zurich 8092, Switzerland; Center for Data Science, New York University, New York, NY 10011, United States; Department of Computer Science, New York University, New York, NY 10012, United States

**Keywords:** artificial intelligence, healthcare, causal inference

## Abstract

The primary practice of healthcare artificial intelligence (AI) starts with model development, often using state-of-the-art AI, retrospectively evaluated using metrics lifted from the AI literature like AUROC and DICE score. However, good performance on these metrics may not translate to improved clinical outcomes. Instead, we argue for a better development pipeline constructed by working backward from the end goal of positively impacting clinically relevant outcomes using AI, leading to considerations of causality in model development and validation, and subsequently a better development pipeline. Healthcare AI should be “actionable,” and the change in actions induced by AI should improve outcomes. Quantifying the effect of changes in actions on outcomes is causal inference. The development, evaluation, and validation of healthcare AI should therefore account for the causal effect of intervening with the AI on clinically relevant outcomes. Using a causal lens, we make recommendations for key stakeholders at various stages of the healthcare AI pipeline. Our recommendations aim to increase the positive impact of AI on clinical outcomes.

Artificial intelligence (AI) holds great promise to improve healthcare. Modern AI excels at modeling complex associations in data to automate clinical workflows, predict patient outcomes, diagnose conditions, recommend interventions, or broaden clinical knowledge.^1–3^ The democratization of AI with open-source software, datasets, and large-scale computing has led to AI innovation in healthcare.^4^ This success, in concert with the availability of healthcare data for research,^5^ has generated significant interest in harnessing AI to improve patient outcomes.^6–8^ So far, the broader success of AI has remained unrealized in healthcare.^9–11^ Here, we highlight that this is partly because improving patient outcomes is not prioritized in the AI development, evaluation, and validation pipeline.

Currently, AI development for healthcare often begins with training state-of-the-art AI methods on a healthcare task, where success is measured using broadly used model performance metrics, like AUROC (Area Under the Receiver Operating Characteristic Curve) and DICE score (harmonic mean of precision and recall, also called F1-Score), on whatever held-out data are at hand. However, clinical validation of AI evaluates the utility of intervening with these models on clinically relevant outcomes. The difference between model evaluation during development and the goal of improving clinically relevant outcomes implies a disconnect. As one example, metrics such as AUROC, unlike decision-analytic measures,^12^ only evaluate performance in a predictive task, disconnected from the effect of intervening with the AI model.^13^ As another example, the target population to which the model will be applied may differ from the held-out population on which the model was retrospectively validated. For instance, predictions may only be available for a subset of clinical units that have been educated about an AI’s use, a form of selection bias.

Consider identifying structural heart disease (SHD) using electrocardiograms (ECGs).^14^ SHD is diagnosed using an echocardiogram (echo), an expensive data modality not available for all patients. ECG is 10 times more prevalent and 20 times less expensive than echo, but human experts cannot identify SHD using ECG alone. There is emerging evidence that AI models can use ECGs to diagnose SHD, turning cheap and common ECGs into a screening tool.^14^ Here, ground truth labels exist only in patients who also received an echo. Thus, the subpopulation of patients with both ECG and an echo constitutes the training and validation data used for model selection. Clinically, the model could be deployed to match the current workflow. That is, the model can run only on patients for whom a clinician has ordered an echo. Used in this way, the model could help avoid unnecessary testing but would not address SHD underdiagnosis. To identify cases that would otherwise be missed, ideally the model would apply to all patients with an ECG, including those who may not have an echo. However, model performance measures like positive predictive value (PPV), on patients with both an ECG and an echo, do not necessarily translate to a high PPV on patients who received an ECG, since prevalence differs between these cohorts.

Understanding how the AI will be integrated into clinical workflows can help identify differences in evaluation versus deployment cohorts and inform model development, such as adjusting for the difference using re-weighting.^15^ Accounting for such population differences during development will reduce the chance that these challenges are only uncovered during prospective validation. An example of prospective validation is a silent trial, where the model output is not available or visible during clinical decision-making but validated on a relevant outcome.^16–18^ For example, a silent validation identified that a population shift due to age distribution differences, proportion of left and right kidneys, and format differences caused an AI predicting obstructive hydronephrosis in infants to deteriorate.^17^ Reviewing differences in populations prior to silent testing could have saved time and resources.

Integrated AI solutions should be *actionable*, ie, improve downstream decision-making and subsequently, patient outcomes.^19^ The effect of AI on such changed decisions is an *intervention* on the patient. Quantifying the effect of such an *intervention* on patient outcomes is causal inference.

Viewing AI solutions as interventions that should improve patient outcomes allows us to reason backward from this goal to enhance the AI development pipeline. We highlight critical considerations to build the “best feasible” AI, ie, interventions most likely to deliver good outcomes considering the effort required to improve the quality of estimates of the AI intervention on patient outcomes.

## Task conception

Prioritizing patient outcomes can organically inform the correct learning task.

## Prospective integration and validation

Ideal evaluation of AI solutions should measure relevant primary endpoints and safety. Other secondary outcomes must be justified in the context of the primary endpoint.

## Model selection and evaluation

Prospective validation of AI, especially Randomized Controlled Trials (RCTs), is expensive. AI development leading up to experimental validation should make the best use of the data at hand for model selection and evaluation. We emphasize 2 forms of evaluation crucial for healthcare AI.

1. Silent trials: Silent trials enable running a model in the background without influencing clinical decision-making. A silent trial that measures a proximal outcome correlated to the target outcome of interest can help quantify model behavior under natural differences from the training conditions without affecting clinical decisions, mitigating safety concerns.
2. Causal off-policy evaluation (OPE)^20^^,^^21^: Retrospective evaluation and model selection should go beyond evaluation on held-out data and simulate the effect of the AI intervention, for example, using causal OPE.

## Model design

AI training and design choices should proactively reduce the burden on evaluations to surface potential obstacles to trial success, such as differences in the training and deployment population, or inadequate handling of statistical biases. AI solutions that seek to improve outcomes through optimizing decisions (eg, recommend treatments) target a causal task and should use methods such as observational causal inference.^22–24^

## Continual monitoring, validation, and improvement

Evolving patient populations and the effect of deploying the AI intervention itself suggest that static models cannot be guaranteed to consistently improve patient outcomes. However, compared to pharmaceutical interventions, AI interventions can provide shorter feedback loops on patient outcomes, enabling improvements in subpopulations for whom the model may currently be underperforming.

**Figure 1.**
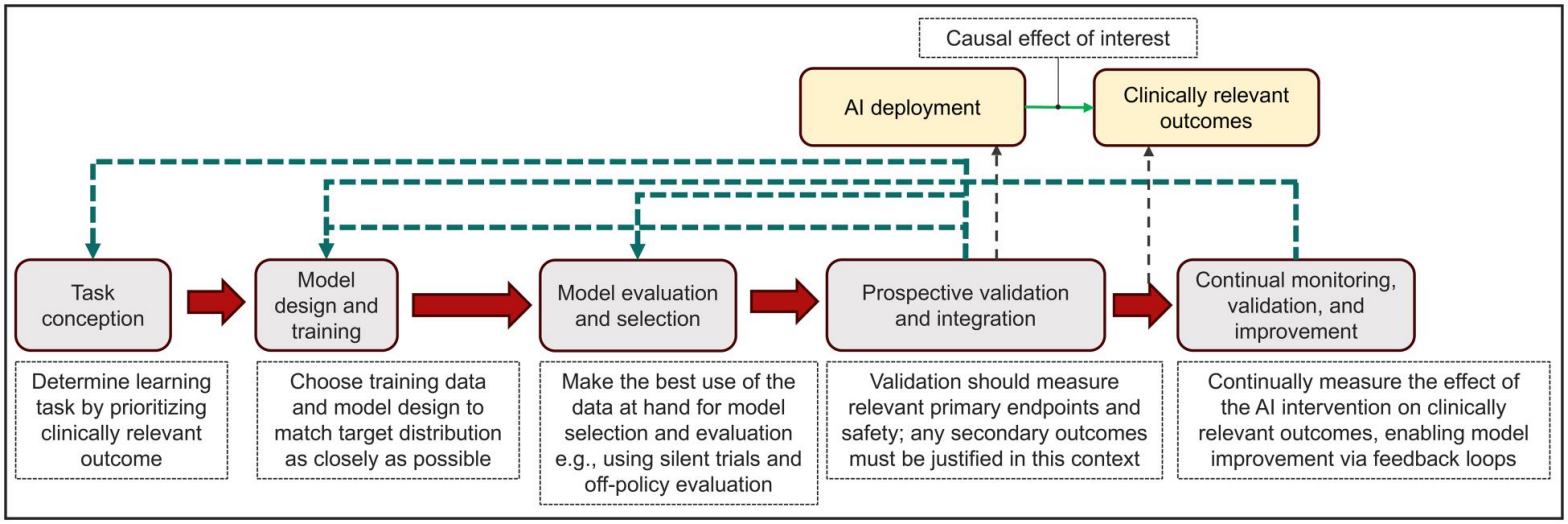
Deploying AI in healthcare is an intervention that can impact clinical outcomes. The figure shows the proposed healthcare AI development pipeline which centers on a causal approach to AI development with the goal of improving clinically relevant outcomes. Knowledge of outcomes of interest should inform relevant endpoints, and hence the task conception, data used for training, model design, the design of silent validation and model selection protocols, and prospective validation. Continual monitoring, validation, and improvement should involve measuring the effect of the AI intervention on clinically relevant outcomes.

Figure 1 summarizes the proposed healthcare AI development pipeline. We identify actionable recommendations for key stakeholders in various phases of the healthcare AI pipeline.

## Recommendations to the AI research community

The AI community has and will likely play a defining role in determining which clinical questions are the focus of potential AI solutions, such as the choice of methods usually employed, early-stage model evaluation prior to silent and prospective validation, and methodological innovation for post-deployment model improvement.

- Task conception: The AI community should consider: What task is important and how to train the AI model to succeed in this task? While the precise task will likely be defined in collaboration with clinicians and patients (and based on the availability of data), it is important to consider that AI solutions are used by humans. Therefore, the AI community should account for the AI solution’s impact on decision-making when defining the task.
- Model selection and evaluation: Moving beyond standard evaluation approaches, healthcare AI developers should consider advanced techniques that account for potential differences in population once deployed. Advanced evaluations such as OPE are currently largely unknown, under-utilized, or not broadly acceptable, despite their utility in quantifying the benefit of AI interventions. We argue for a broader dissemination and acceptance of such methods among clinical model developers and peer reviewers. Nonetheless, these methods require checking sensitivity to assumptions that may not hold in practice.^20^
- Model design: Healthcare AI developers should consider whether the candidate training data are close to the deployment population and of reasonable quality, ie, with acceptable levels of statistical bias. This may be measured according to multiple objectives, eg, equal subgroup performance if desirable. Model designs that capture generalizable associations (as opposed to spurious correlations)^25^ are more likely to perform well when the training and deployment populations differ. AI models should be trained to proactively account for biases such as missingness and censoring, for example, using survival analysis,^26–29^ including accounting for any equity goals.^30–33^
- Continual monitoring, validation, and improvement: New research for continual model monitoring, validation, and improvement based on improvement in patient outcomes is necessary.

## Recommendations to the causal inference, implementation science, and trial design community

There is a prolific community focused on methods for drawing causal inferences from observational and experimental data^23^^,^^24^^,^^34^ that could inform advances in prospective integration and validation of AI models, and experiment design for continual monitoring and model improvement, primarily to bridge the current methodological chasm.

- Prospective integration and validation: We surface key lessons learned by this community to enable rigorous evaluation of AI interventions: appropriate experimental design and evaluating patient-focused endpoints are crucial. The effect of an AI intervention on patient outcomes may be heterogeneous and should be quantified using robust analysis. When randomization is not possible, observational causal inference methods may be useful but must be used carefully, allowing for unmeasured confounding, such as using sensitivity analysis.^35–38^ We also encourage the causal inference community to engage closely with AI development and consider clinical AI interventions as worthy of evaluation.
- Continual monitoring, validation, and improvement: To enable continual validation, dynamic trials, such as adaptive trial designs,^39^ are valuable, but may not keep up with AI technological improvements. In addition, new validation and AI improvement strategies such as safe micro-randomization may be necessary.^40^

## Recommendations for publishing guidelines of healthcare AI

Publication guidelines indicate minimal reporting requirements acceptable to the scientific community. The current reporting guidelines for AI development and evaluation may be underspecified to improve the impact of AI solutions and hence benefit from further informing reporting guidelines for model design, and the development of new guidelines for post-deployment monitoring, validation, and improvement.

- Model design: For example, TRIPOD+AI,^41^ a minimal reporting guideline for initial model development does require a discussion on how the AI intervention will be incorporated into the clinical pathway and inform decision-making. While the guidance also asks for modeling rationale, they do not explicitly ask for a report of how the clinical use justifies model design and evaluation choices.^42^ DECIDE-AI,^43^ CONSORT-AI,^44^ and SPIRIT-AI^45^ guidelines require reporting design and evaluation choices in the context of the clinical use case. We recommend that these discussions, specifically tying the clinical use case to model design choices be incorporated in the TRIPOD+AI checklist.
- Continual monitoring, validation, and improvement: New publishing guidelines and post-market regulatory policies are necessary for reporting and emphasizing improved infrastructure for continual monitoring, validation, and improvement of AI interventions.^46^ Such regulations should reconcile with ethical tensions in consent-based publishing and the ethics of improving care for all.
- Recommendations to healthcare institutions: Healthcare institutions can benefit from improved healthcare AI pipelines if there is adequate infrastructure in place to enable safe AI integration, deployment, monitoring, and improvement.
- Continual monitoring, validation, and improvement: Healthcare institutions should prioritize compute and data infrastructure, and personnel training for continual monitoring for safety and efficacy,^47–49^ beyond monitoring performance metrics and enabling feedback of AI-supported actions on patient outcomes.

These recommendations surface the need for tight-knit collaborations between several stakeholders to address multiple aspects of the healthcare AI pipeline, with a progressively higher number of involved stakeholders over the stages of the healthcare AI pipeline.

The United States Food and Drug Administration (FDA) has consolidated a comprehensive set of “Good Machine Learning Practices” (GMLP) for the healthcare AI pipeline.^50^ We discuss a few key guiding principles from this practice in the context of the causal lens. The GMLP recommends that “Data Sets Are Representative of the Intended Patient Population” and “Testing Demonstrates Device Performance During Clinically Relevant Conditions.”

Since AI solutions are causal interventions, not only is representativeness a necessity, but there is also a need to proactively design AI to prevent any anticipated mismatch in training and deployment populations and enhance model design if such mismatch is not preventable. Even after establishing the desirable efficacy of an AI intervention, several post-deployment steps are necessary to monitor and improve the effect on patient outcomes. For example, GMLP guidelines recommend that “Deployed Models Are Monitored for Performance and Re-training Risks Are Managed.” A focus on model performance alone is insufficient and the operationalization of the GMLP principles in the “Pre-determined Change Protocol Plans” should be grounded in how the AI intervention affects clinically relevant outcomes, in addition to “device performance” throughout its life cycle, including assessing efficacy-risk tradeoffs.

Furthermore, there may be scenarios where an existing AI model is applied to a new setting, ie, “off-label use” of AI due to infeasibility of model development.^51^ Here, causal thinking is equally important in evaluating the appropriateness. Careful retrospective evaluation with attention to patient population differences and primary outcomes can save time and effort if the model does not inform new uses.

To deliver on the promise of healthcare AI, considering the desired causal impact of an AI solution on downstream decision-making and clinically relevant outcomes is imperative. There are additional cognitive, research, and resource costs to achieving the desirable causal impact and may entail longer and iterative model development time, reliance on more sophisticated model selection methods, and pre-deployment user studies for evaluating impact on decision-making. However, such considerations will ultimately increase the chances of success of models once deployed. This work highlights several desirable changes in how the healthcare AI research community should approach model development, evaluation, and validation. Implementation science, causal inference, and trial design communities can lend practical insights for validating AI interventions, and the need for new healthcare infrastructure to support such developments. Additionally, several advances in regulatory, ethical, and publication guidelines for healthcare AI while upholding bioethical principles are necessary.

## Data Availability

There are no new data associated with this article.

## References

[1] Rajpurkar P , Chen E, Banerjee O, et al AI in health and medicine. Nat Med. 2022;28:31-38. 35058619 10.1038/s41591-021-01614-0

[2] Ghassemi M , Naumann T, Schulam P, et al A review of challenges and opportunities in machine learning for health. AMIA Summits Transl Sci Proc. 2020;2020:191-200. 32477638 PMC7233077

[3] Chen IY , Joshi S, Ghassemi M, et al Probabilistic machine learning for healthcare. Annu Rev Biomed Data Sci. 2021;4:393-415. 34465179 10.1146/annurev-biodatasci-092820-033938 PMC13234866

[4] Dean J. A golden decade of deep learning: computing systems & applications. Daedalus. 2022;151:58-74.

[5] Alberto IRI , Alberto NRI, Ghosh AK, et al The impact of commercial health datasets on medical research and health-care algorithms. Lancet Digit Health. 2023;5:e288-e294. 37100543 10.1016/S2589-7500(23)00025-0 PMC10155113

[6] Wiens J , Saria S, Sendak M, et al Do no harm: a roadmap for responsible machine learning for health care. Nat Med. 2019;25:1337-1340. 31427808 10.1038/s41591-019-0548-6

[7] Topol EJ. High-performance medicine: the convergence of human and artificial intelligence. Nat Med. 2019;25:44-56. 30617339 10.1038/s41591-018-0300-7

[8] Baldi P. Deep learning in biomedical data science. Annu Rev Biomed Data Sci. 2018;1:181-205.

[9] Kelly CJ , Karthikesalingam A, Suleyman M, et al Key challenges for delivering clinical impact with artificial intelligence. BMC Med. 2019;17:195. 31665002 10.1186/s12916-019-1426-2 PMC6821018

[10] Panch T , Mattie H, Celi LA. The “inconvenient truth” about AI in healthcare. NPJ Digit Med. 2019;2:77. 31453372 10.1038/s41746-019-0155-4 PMC6697674

[11] Petersson L , Larsson I, Nygren JM, et al Challenges to implementing artificial intelligence in healthcare: a qualitative interview study with healthcare leaders in Sweden. BMC Health Serv Res. 2022;22:850. 35778736 10.1186/s12913-022-08215-8 PMC9250210

[12] Vickers AJ , Elkin EB. Decision curve analysis: a novel method for evaluating prediction models. Med Decis Making. 2006;26:565-574. 17099194 10.1177/0272989X06295361 PMC2577036

[13] van Amsterdam WAC , van Geloven N, Krijthe JH, et al When accurate prediction models yield harmful self-fulfilling prophecies. arXiv [stat.ME]. 2023, preprint: not peer reviewed. 10.1016/j.patter.2025.101229 PMC12010445 40264961

[14] Jing L , Finer J, Hartzel D, et al Abstract 14647: EchoNext: an ECG-based deep learning model to detect structural heart disease. Circulation. 2023;148. 10.1161/circ.148.suppl_1.14647

[15] Jethani N , Puli A, Zhang H, et al New-onset diabetes assessment using artificial intelligence-enhanced electrocardiography. arXiv [cs.LG]. 2022, preprint: not peer reviewed.

[16] van der Vegt AH , Scott IA, Dermawan K, et al Implementation frameworks for end-to-end clinical AI: derivation of the SALIENT framework. J Am Med Inform Assoc. 2023;30:1503-1515. 37208863 10.1093/jamia/ocad088 PMC10436156

[17] Kwong JCC , Erdman L, Khondker A, et al The silent trial—the bridge between bench-to-bedside clinical AI applications. Front Digit Health. 2022;4:929508. 36052317 10.3389/fdgth.2022.929508 PMC9424628

[18] Tonekaboni S , Morgenshtern G, Assadi A, et al How to validate machine learning models prior to deployment: silent trial protocol for evaluation of real-time models at ICU. In: Flores G, Chen GH, Pollard T, et al, eds. Proceedings of the Conference on Health, Inference, and Learning. Vol. 174. PMLR; 2022:169-182.

[19] Ehrmann DE , Joshi S, Goodfellow SD, et al Making machine learning matter to clinicians: model actionability in medical decision-making. NPJ Digit Med. 2023;6:7. 36690689 10.1038/s41746-023-00753-7 PMC9871014

[20] Gottesman O , Johansson F, Komorowski M, et al Guidelines for reinforcement learning in healthcare. Nat Med. 2019;25:16-18. 30617332 10.1038/s41591-018-0310-5

[21] Uehara M , Shi C, Kallus NA. Review of off-policy evaluation in reinforcement learning. arXiv [stat.ML]. 2022, preprint: not peer reviewed.

[22] Lin L , Sperrin M, Jenkins DA, et al A scoping review of causal methods enabling predictions under hypothetical interventions. Diagn Progn Res. 2021;5:3. 33536082 10.1186/s41512-021-00092-9 PMC7860039

[23] Pearl J. Causality. Cambridge University Press; 2009.

[24] Hernan MA , Robins JM. Causal Inference. CRC Press; 2019.

[25] Puli A , Zhang LH, Oermann E, et al Out-of-distribution generalization in the presence of nuisance-induced spurious correlations. In: *International Conference on Learning Representations*. 2022.

[26] Ranganath R , Perotte A. Deep survival analysis. In: *Proceedings of the First Machine Learning for Healthcare Conference*. Vol. 56. PMLR; 2016:101-114.

[27] Miscouridou X , Perotte A, Elhadad N, et al Deep survival analysis: nonparametrics and missingness. MLHC. 2018;85:244-256.

[28] Lee C , Zame W, Yoon J, et al DeepHit: a deep learning approach to survival analysis with competing risks. Proc AAAI Conf Artif Intell. 2018;32. 10.1609/aaai.v32i1.11842

[29] Goldstein M , Han X, Puli A, et al X-CAL: explicit calibration for survival analysis. Adv Neural Inf Process Syst. 2020;33:18296-18307. 34017160 PMC8132615

[30] Chen IY , Pierson E, Rose S, et al Ethical machine learning in healthcare. Annu Rev Biomed Data Sci. 2021;4:123-144. 34396058 10.1146/annurev-biodatasci-092820-114757 PMC8362902

[31] Barocas S , Hardt M, Narayanan A. Fairness and Machine Learning: Limitations and Opportunities. MIT Press; 2023.

[32] Rajkomar A , Hardt M, Howell MD, et al Ensuring fairness in machine learning to advance health equity. Ann Intern Med. 2018;169:866-872. 30508424 10.7326/M18-1990 PMC6594166

[33] Chen RJ , Wang JJ, Williamson DFK, et al Algorithmic fairness in artificial intelligence for medicine and healthcare. Nat Biomed Eng. 2023;7:719-742. 37380750 10.1038/s41551-023-01056-8 PMC10632090

[34] Madon T , Hofman KJ, Kupfer L, et al Public health. Implementation science. Science. 2007;318:1728-1729. 18079386 10.1126/science.1150009

[35] Robins JM , Rotnitzky A, Scharfstein DO. Sensitivity analysis for selection bias and unmeasured confounding in missing data and causal inference models. In: Halloran ME, Berry D, eds. Statistical Models in Epidemiology, the Environment, and Clinical Trials. Springer New York; 2000:1-94.

[36] Hill AB. The environment and disease: association or causation? Proc R Soc Med. 1965;58:295-300. 14283879 10.1177/003591576505800503 PMC1898525

[37] Liu W , Kuramoto SJ, Stuart EA. An introduction to sensitivity analysis for unobserved confounding in nonexperimental prevention research. Prev Sci. 2013;14:570-580. 23408282 10.1007/s11121-012-0339-5 PMC3800481

[38] Chernozhukov V , Cinelli C, Newey W, Sharma A, Syrgkanis V. Long Story Short: Omitted Variable Bias in Causal Machine Learning. No. w30302. National Bureau of Economic Research; 2022.

[39] Pallmann P , Bedding AW, Choodari-Oskooei B, et al Adaptive designs in clinical trials: why use them, and how to run and report them. BMC Med. 2018;16:29. 29490655 10.1186/s12916-018-1017-7 PMC5830330

[40] Horwitz LI , Kuznetsova M, Jones SA. Creating a learning health system through rapid-cycle, randomized testing. N Engl J Med. 2019;381:1175-1179. 31532967 10.1056/NEJMsb1900856

[41] Collins GS , Moons KGM, Dhiman P, et al TRIPOD+AI statement: updated guidance for reporting clinical prediction models that use regression or machine learning methods. BMJ. 2024;385:e078378. 38626948 10.1136/bmj-2023-078378 PMC11019967

[42] van Amsterdam WAC , Cinà G, Didelez V, et al Prognostic models for decision support need to report their targeted treatments and the expected changes in treatment decisions. BMJ. 2024;385:e078378.

[43] Vasey B , Novak A, Ather S, et al DECIDE-AI: a new reporting guideline and its relevance to artificial intelligence studies in radiology. Clin Radiol. 2023;78:130-136. 36639172 10.1016/j.crad.2022.09.131

[44] Liu X , Cruz Rivera S, Moher D, SPIRIT-AI and CONSORT-AI Working Group, et al Reporting guidelines for clinical trial reports for interventions involving artificial intelligence: the CONSORT-AI extension. Nat Med. 2020;26:1364-1374. 32908283 10.1038/s41591-020-1034-x PMC7598943

[45] Cruz Rivera S , Liu X, Chan A-W, SPIRIT-AI and CONSORT-AI Consensus Group, et al Guidelines for clinical trial protocols for interventions involving artificial intelligence: the SPIRIT-AI extension. Nat Med. 2020;26:1351-1363. 32908284 10.1038/s41591-020-1037-7 PMC7598944

[46] Ayers JW , Desai N, Smith DM. Regulate artificial intelligence in health care by prioritizing patient outcomes. JAMA. 2024;331:639-640. 38285467 10.1001/jama.2024.0549

[47] Youssef A , Pencina M, Thakur A, et al External validation of AI models in health should be replaced with recurring local validation. Nat Med. 2023;29:2686-2687. 37853136 10.1038/s41591-023-02540-z

[48] Corbin CK , Maclay R, Acharya A, et al DEPLOYR: a technical framework for deploying custom real-time machine learning models into the electronic medical record. J Am Med Inform Assoc. 2023;30:1532-1542. 37369008 10.1093/jamia/ocad114 PMC10436147

[49] Sendak MP , Gao M, Brajer N, et al Presenting machine learning model information to clinical end users with model facts labels. NPJ Digit Med. 2020;3:41. 10.1038/s41746-020-0253-3 32219182 PMC7090057

[50] U.S. Food and Drug Administration. *Good Machine Learning Practice for Medical Device Development: Guiding Principles*. U.S. Food and Drug Administration; 2023. Accessed 16 August 2024. https://www.fda.gov/medical-devices/software-medical-device-samd/good-machine-learning-practice-medical-device-development-guiding-principles

[51] Krishnamoorthy M , Sjoding MW, Wiens J. Off-label use of artificial intelligence models in healthcare. Nat Med. 2024;30:1525-1527. 38519769 10.1038/s41591-024-02870-6

